# Group II Metabotropic Glutamate Receptors Depress Synaptic Transmission onto Subicular Burst Firing Neurons

**DOI:** 10.1371/journal.pone.0045039

**Published:** 2012-09-11

**Authors:** Michael Kintscher, Jörg Breustedt, Stéphanie Miceli, Dietmar Schmitz, Christian Wozny

**Affiliations:** 1 Neuroscience Research Center of the Charité – Universitätsmedizin Berlin, Berlin, Germany; 2 NeuroCure Cluster of Excellence, Charité – Universitätsmedizin Berlin, Berlin, Germany; Consejo Superior de Investigaciones Cientificas - Instituto Cajal, Spain

## Abstract

The subiculum (SUB) is a pivotal structure positioned between the hippocampus proper and various cortical and subcortical areas. Despite the growing body of anatomical and intrinsic electrophysiological data of subicular neurons, modulation of synaptic transmission in the SUB is not well understood. In the present study we investigated the role of group II metabotropic glutamate receptors (mGluRs), which have been shown to be involved in the regulation of synaptic transmission by suppressing presynaptic cAMP activity. Using field potential and patch-clamp whole cell recordings we demonstrate that glutamatergic transmission at CA1-SUB synapses is depressed by group II mGluRs in a cell-type specific manner. Application of the group II mGluR agonist (2S,1′R,2′R,3′R)-2-(2, 3-dicarboxycyclopropyl)glycine (DCG-IV) led to a significantly higher reduction of excitatory postsynaptic currents in subicular bursting cells than in regular firing cells. We further used low-frequency stimulation protocols and brief high-frequency bursts to test whether synaptically released glutamate is capable of activating presynaptic mGluRs. However, neither frequency facilitation is enhanced in the presence of the group II mGluR antagonist LY341495, nor is a test stimulus given after a high-frequency burst. In summary, we present pharmacological evidence for presynaptic group II mGluRs targeting subicular bursting cells, but both low- and high-frequency stimulation protocols failed to activate presynaptically located mGluRs.

## Introduction

The subiculum (SUB) is the main output region of the hippocampal formation and functions as the major interface between the hippocampus proper and various cortical and subcortical regions. The SUB receives direct synaptic input from parahippocampal regions, but also indirectly via the well-known trisynaptic pathway of the hippocampus [1], [2]. Due to its pivotal position in the hippocampal circuitry it is not surprising that the SUB has been shown to be implicated in certain diseases like epilepsy and schizophrenia with pathological features of neuronal hyperexcitability, enhanced glutamatergic neurotransmission and altered neuronal morphology [3]–[6]. Many of the drugs currently used to treat hyperexcitability disorders either inhibit glutamatergic transmission directly or strengthen inhibitory transmission to fine-tune the excitation-inhibition balance [7], [8].

**Figure 1:**
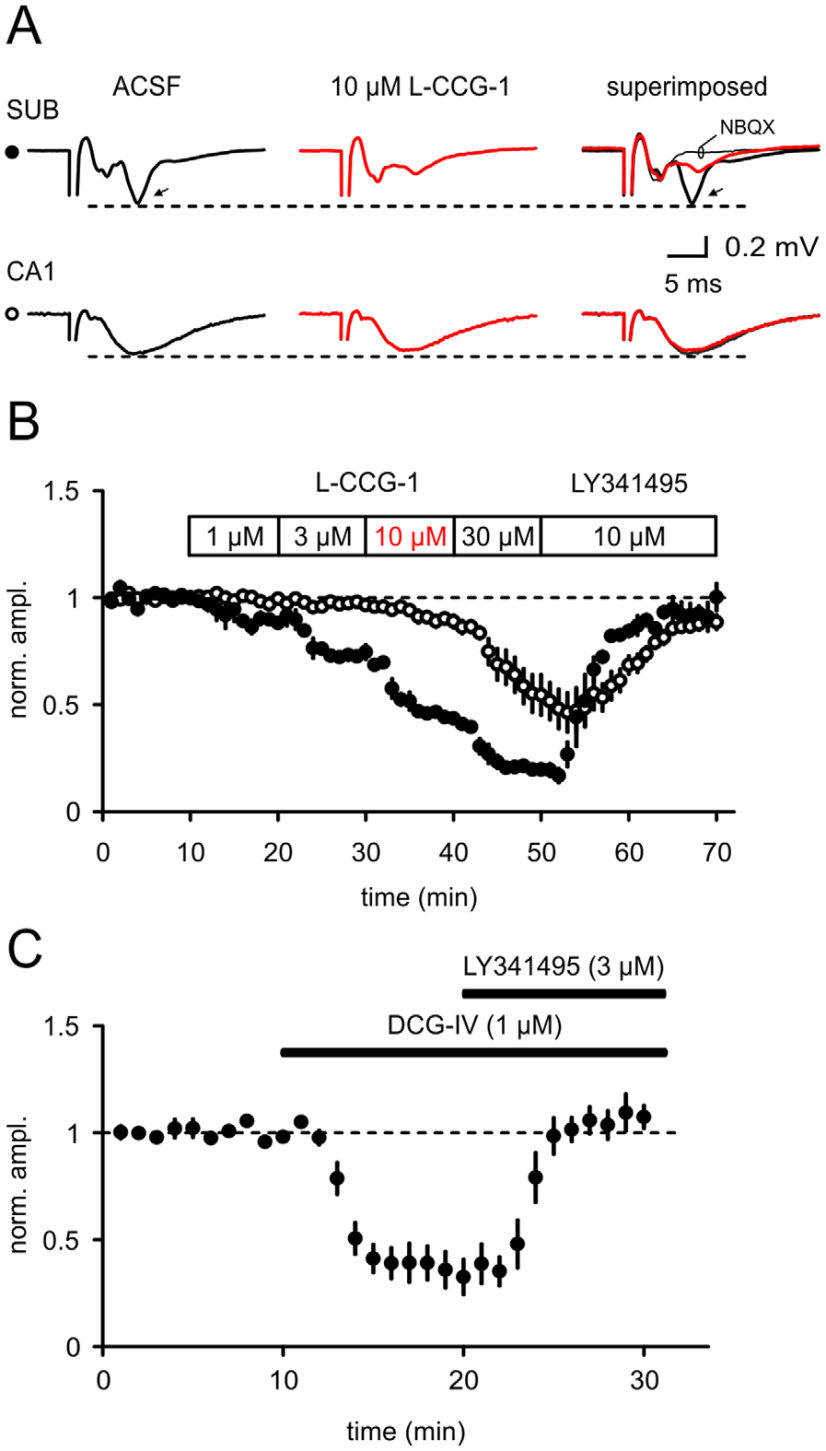
The group II agonist L-CCG-1 differently affects synaptic transmission in areas CA1 and SUB. (A, B) Field potential recordings were performed and the group II agonist L-CCG 1 was bath-applied in various concentrations. Field potentials were depressed in a concentration-dependent manner (SUB: n = 4; CA1: n = 5). (C) DCG-IV (1 µM) suppressed fEPSP in the SUB to a similar extent as observed with 10 µM L-CCG-1 (SUB: n = 5; all in presence of the NMDA receptor antagonist APV, 50 µM).

Metabotropic glutamate receptors (mGluRs) have important roles in regulating synaptic transmission [9] by providing a negative feedback of glutamatergic transmission at central neurons [10]–[13]. Pharmacological activation of mGluRs therefore provides a treatment option for hyperexcitability disorders as epilepsy or schizophrenia [14], [15]. The mGluRs are a family of G protein coupled receptors comprised of eight subtypes (mGluR1–8) classified into 3 subgroups I, II and III. In general, group II and III mGluRs appear to negatively modulate excitatory neurotransmission [16]. In the hippocampus, mGluRs are differentially expressed in hippocampal subfields. Group II mGluR agonists are used to discriminate mossy fiber input from associational–commissural (A/C) input in area CA3 as mossy fiber synapses express presynaptic group II metabotropic glutamate receptors (mGluRs), but A/C synapses do not [17]. Schaffer collateral synapses also express few group II mGluRs [18], [19]. In the present study, we examined the effects of the specific group II mGluR agonists, (2S,1′S,2′S)-2-(2-carboxycyclopropyl)glycine (L-CCG-1) and (2S,1′R,2′R,3′R)-2-(2, 3-dicarboxycyclopropyl)glycine (DCG-IV), in the SUB and compared these findings with the application of these drugs in areas CA1 and CA3. We show that group II mGluRs activation differently affects synaptic transmission in these three different brain regions of the hippocampal formation. At CA1-SUB synapses excitatory postsynaptic responses are reduced in the presence of the mGluR agonists in a target-specific manner.

**Figure 2:**
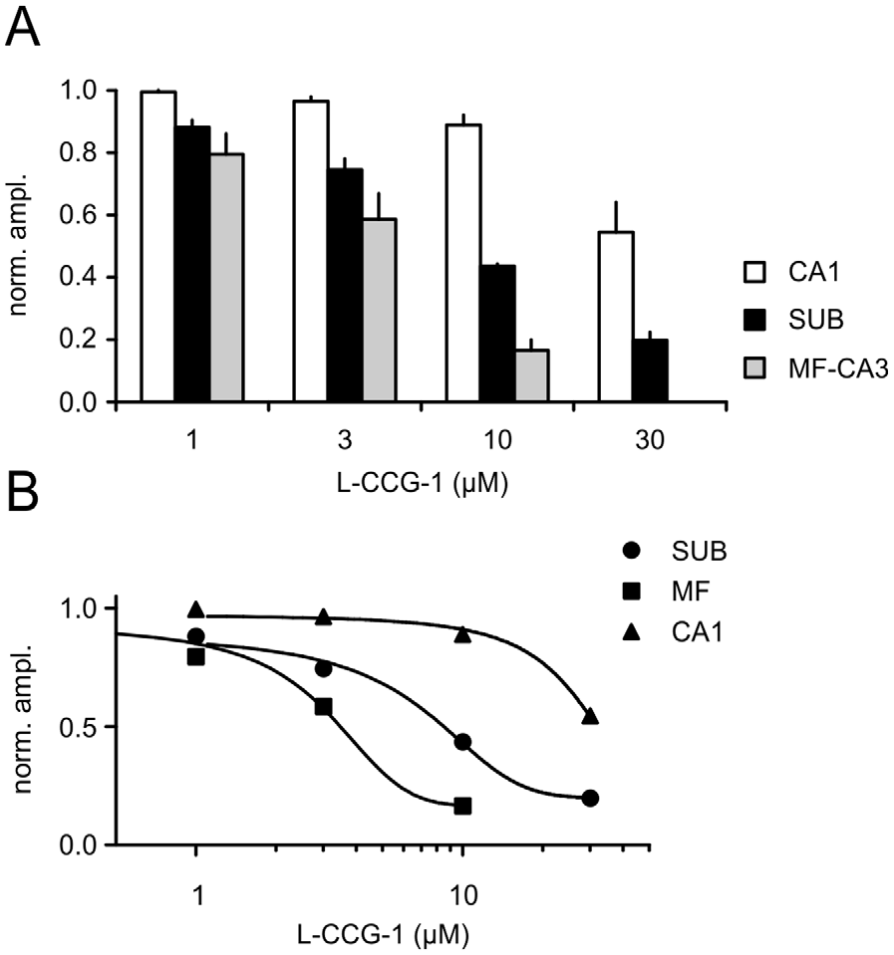
L-CCG-1 differentially depresses glutamatergic transmission in three different brain regions of the hippocampal formation. (A)Summary bar diagram of the effects of different concentrations of L-CCG-1 in areas CA1, CA3 (MF) and SUB (SUB: n = 4; CA1: n = 5; MF-CA3: n = 6). (B) Data were fitted to a sigmoidal function and a dose-response curve is given for the SUB, MF-CA3 and CA1. EC50 values were estimated to 7 µM, 3 µM and 28 µM for the SUB, MF-CA3 and CA1, respectively. Error bars are not shown for clarity.

## Methods and Materials

### Ethics Statement

Animal husbandry and experimental intervention were performed according to the German animal welfare act and the European Council Directive 86/609/EEC regarding the protection of animals used for experimental and other scientific purposes. All animal maintenance was performed in accordance to national and international guidelines and was approved by local federal state authorities, Landesamt fuer Gesundheit und Soziales (LAGeSo), Berlin, Germany (T0073/04).

### Slice Preparation and Electrophysiology

Wistar rats (3–5 weeks, male and female) were decapitated under deep ether or isoflurane anaesthesia and the brains were quickly removed. 300 µm thick rat brain slices were prepared in sucrose-based ACSF (in mM): NaCl 87, NaH_2_PO_4_ 1.25, KCl 2.5, NaHCO_3_ 26, MgCl_2_ 7, CaCl_2_ 0.5, sucrose 75 and Glucose 25. After half an hour of incubation at 35–37°C slices were transferred to physiological ACSF solution (containing in mM: NaCl 124, NaH_2_PO_4_ 1.25, KCl 3, MgSO_4_ 1.3, CaCl_2_ 2.5, NaHCO_3_ 26, glucose 10, saturated with 95% O_2_ and 5% CO_2_ at a pH of 7.4).

Whole-cell voltage-clamp and field excitatory postsynaptic potential (fEPSP) recordings were performed with an Axopatch 700B amplifier (Axon Instruments, Union City, CA, USA). Data were recorded and filtered at 2–4 kHz, digitized (National Instruments BNC-2090) at 5–10 kHz and analysed with custom-made software in IGOR Pro (WaveMetrics Inc., OR, USA).

**Figure 3:**
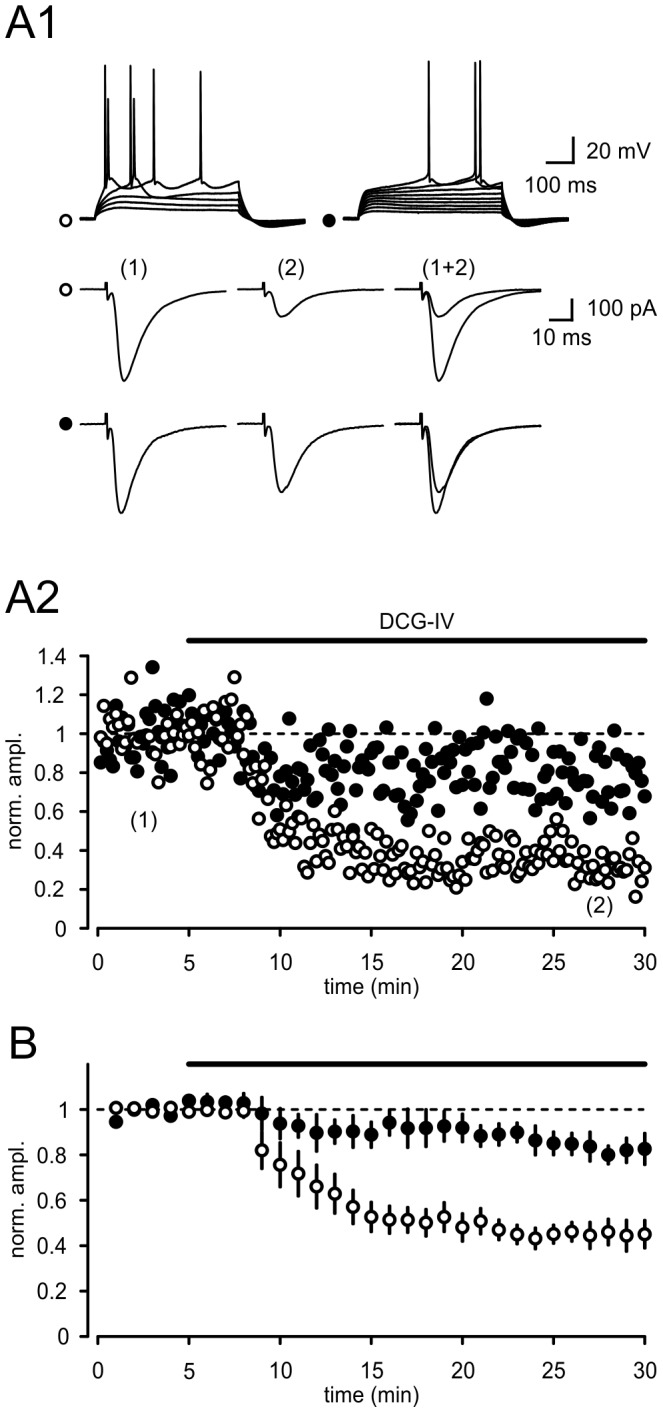
DCG-IV selectively inhibits synaptic transmission onto burst firing neurons. (A1) Discharge pattern of two different types of subicular pyramidal cells. Open circle: bursting neurons; closed circle: regular firing cells. (A2) Time courses of EPSC amplitudes in representative examples of a bursting and a regular firing cell before and after application of DCG-IV (1–2 µM). (B) Averaged time course for both cell types (BURST: n = 7; REG: n = 5).

Low-resistance patch pipettes filled with ACSF were used for field potential recordings and stimulation of afferent fibres. The subiculum has three layers: (1) a molecular layer containing interneurons and dendrites of pyramidal neurons, (2) an pyramidal cell layer containing the somata of pyramidal neurons, and (3) a polymorphic layer (for review see [20]). Subicular neurons are less densely packed than pyramidal neurons in area CA1. For CA1-subiculum field experiments recordings were done in the middle third of the subiculum at the border between the pyramidal cell layer and the polymorphormic layer and fEPSPs were evoked by alvear stimulation at a frequency of 0.05 Hz. This location has been found to give a relatively large fEPSP in comparison to relatively small signals obtained in the molecular layer (data not shown). The waveform of the subicular fEPSP can be complex due to the diverging CA1 fiber tract and the distribution of subicular neurons along the alvear-fissural axis. In a subset of experiments synaptic transmission was blocked by di-2,3-dioxo-6-nitro-1,2,3,4 tetrahydrobenzo[f]quinoxaline-7-sulfonamide disodium salt (NBQX, 25 µM) to confirm that the measured amplitude (second major negative deflection after stimulus artefact) was not contaminated by a repetitive fiber volley (Fig. 1A). For mossy fiber-CA3 fEPSPs the stimulation electrode was placed in the hilus of the dentate gyrus and field potentials were recorded in stratum lucidum of CA3. For CA1 fEPSPs Schaffer collaterals in CA3 were stimulated and field potentials were recorded in the stratum radiatum of CA1.

Patch-clamp recordings of subicular pyramidal neurons were done in the middle portion of the SUB, which receives synaptic input from the middle subfield of CA1 (with respect to the proximo-distal axis of each region). Single cell recordings were performed in whole cell patch-clamp mode at room temperature. Patch-clamp electrodes (2–5 MΩ) were filled with (in mM): K-gluconate 135, Hepes 10, Mg-ATP 2, KCl 20, EGTA 0.2, pH was adjusted to 7.2 with KOH. Depolarising current steps of 200 up to 1000 ms duration were applied to characterize the cells’ discharge behaviour. Excitatory postsynaptic currents were recorded at –60 mV and were evoked by alvear stimulation. Whole-cell recordings were performed in the presence of the GABA_A_ receptor-antagonists gabazine (SR 95531, 1 µM, purchased from Sigma-Aldrich, Taufkirchen, Germany) and 4 mM MgSO_4_ and CaCl_2_. Paired-pulse facilitation (EPSC2/EPSC1) was investigated by analysing the ratio of the second to the first synaptic response. The coefficient of variation (CV) was calculated as (CV)^−2^ = (standard deviation of EPSCs)^−2^/(mean of EPSCs)^−2^ for a period of 5 minutes before and 25 to 30 minutes after wash-in of DCG-IV. The agonist concentration-response curves were fitted to a sigmoidal function to obtain the EC50 values. The top asymptote was constrained to baseline values, the lower asymptote to less than 20% of the baseline values (GraphPad Software, Prism, La Jolla, USA).

**Figure 4:**
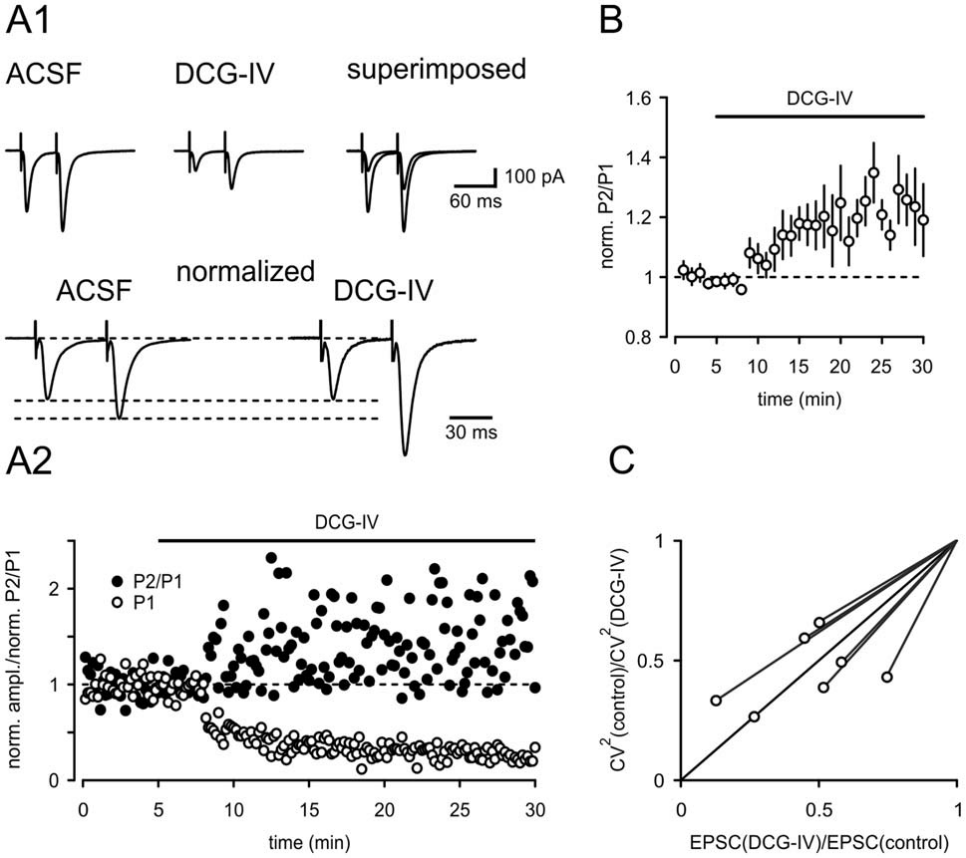
DCG-IV acts presynaptically onto burst firing neurons. (A1 and A2) EPSCs recorded in bursting neurons in response to paired-pulse stimulation before and in the presence application of DCG-IV. Representative example is shown in A1 and A2. The paired-pulse ratio is significantly increased after chemical activation of group II mGlu receptors. A summary of seven experiments is shown in (B). (C) Analysis of the squared coefficient of variation indicates a presynaptic mechanism by which DCG-IV exerts its action.

**Figure 5:**
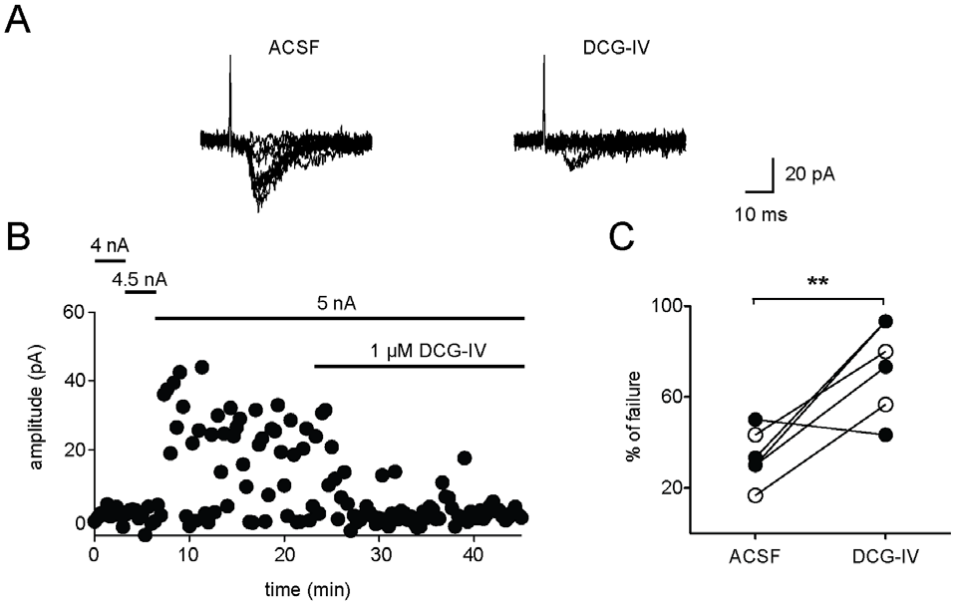
DCG-IV increases failure ratio during minimal stimulation. (A) Representative experiment for a burst firing neuron specific minimal stimulation. Overlays of 10 individual sweeps each are shown for control (ACSF) and 5 min after wash-in of DCG-IV (1 µM). (B) Minimal stimulation strength was achieved by stepwise increase of extracellular current injection by 0.5 nA. After establishing a stable EPSC/failure ratio for at least 10 min DCG-IV was applied. (C) Summary of all experiments showed a significant increase in the percentage of failure in the presence of DCG-IV (n = 6). Open circles represent experiments in the presence of the NMDA receptor antagonist APV (50 µM; for details see text).

For glutamate uncaging experiments 200 µM MNI-caged L-glutamate was used (purchased from Tocris, Biozol, Eching, Germany). The uncaging setup is equipped with a modified pulsed UV laser (355 nm) and the optical system has been adjusted to have an effective light spot diameter of ∼ 20 µm in the focal plane [21], [22]. Glutamate uncaging was performed near the soma of the target cell. Laser flash duration was set to 2 to 3 ms.

Data were expressed as means ± S.E.M (occasionally binned to 1 minute time points). Statistical comparison was performed by applying Student’s *t*-test (Excel, Microsoft) unless otherwise stated. Significance level was set to p<0.05.

## Results

### Effects of mGluR Group II Agonist L-CCG-I on Synaptic Transmission in Areas CA1, CA3 and SUB

We first tested the ability of the mGluR group II agonist L-CCG-1 to inhibit synaptic transmission in two adjacent regions of the hippocampus. In area CA1 concentrations of 1 and 3 µM LCCG-1 did not significantly inhibit field potentials recorded in stratum radiatum (1 µM 1.00±0.01, values normalized to control conditions; 3 µM 0.97±0.01, one-way ANOVA followed by Bonferroni’s post-hoc test, 1 µM tested against 3 µM, p>0.05, n = 5, Fig. 1). Higher concentrations suppressed fEPSP in a dose-dependent manner (10 µM 0.89±0.03, student’s t-test vs. control values: p = 0.03; 30 µM 0.55±0.10, student’s t-test vs. control values: p<0.01; one-way ANOVA followed by Bonferroni’s post-hoc test, 1 µM tested against 30 µM, 3 µM vs. 30 µM and 10 µM vs. 30 µM: p<0.001, Fig. 1). The mGluR group II antagonist LY341495 only partially reversed this blockade (LY 341495 0.89±0.04, n = 5, Fig. 1) indicating a non-specific effect of 30 µM L-CCG-1 in area CA1.

In sharp contrast to the situation in area CA1, application of L-CCG-1 induced a significant decrease of field responses at CA1-SUB synapses. A low concentration of only 1 µM L-CCG-1 already caused a significant reduction of synaptic transmission within the subicular region (1 µM 0.88±0.02; 3 µM 0.75±0.04; 10 µM 0.44±0.01; 30 µM 0.20±0.03; 10 µM LY 341495 0.95±0.07; one-way ANOVA followed by Bonferroni’s post-hoc test: p<0.05 for 1 µM vs. 3 µM and p<0.001 for all other columns tested; n = 4, Fig. 1). In another set of experiments we compared the effects of mGluR II activation within area CA3 (mossy fiber stimulation) and the subiculum. The effects at the hippocampal mossy fiber synapses were slightly more pronounced in comparison to the subiculum (1 µM 0.79±0.07, n = 5; 3 µM 0.59±0.08, n = 6; 10 µM 0.17±0.03, n = 6; one-way ANOVA followed by Bonferroni’s post-hoc test: p<0.001 for 1 µM vs. 10 µM and 3 µM vs. 10 µM; comparison 10 µM SUB vs 10 µM MF: p<0.05; Fig. 2). A full dose-response dependency is illustrated in Figure 2. From these dose-response curves we calculated the EC50 values for the SUB of 7 µM, for the MF of 3 µM and for CA1 of 28 µM (for details see Methods; Fig. 2B).

**Figure 6:**
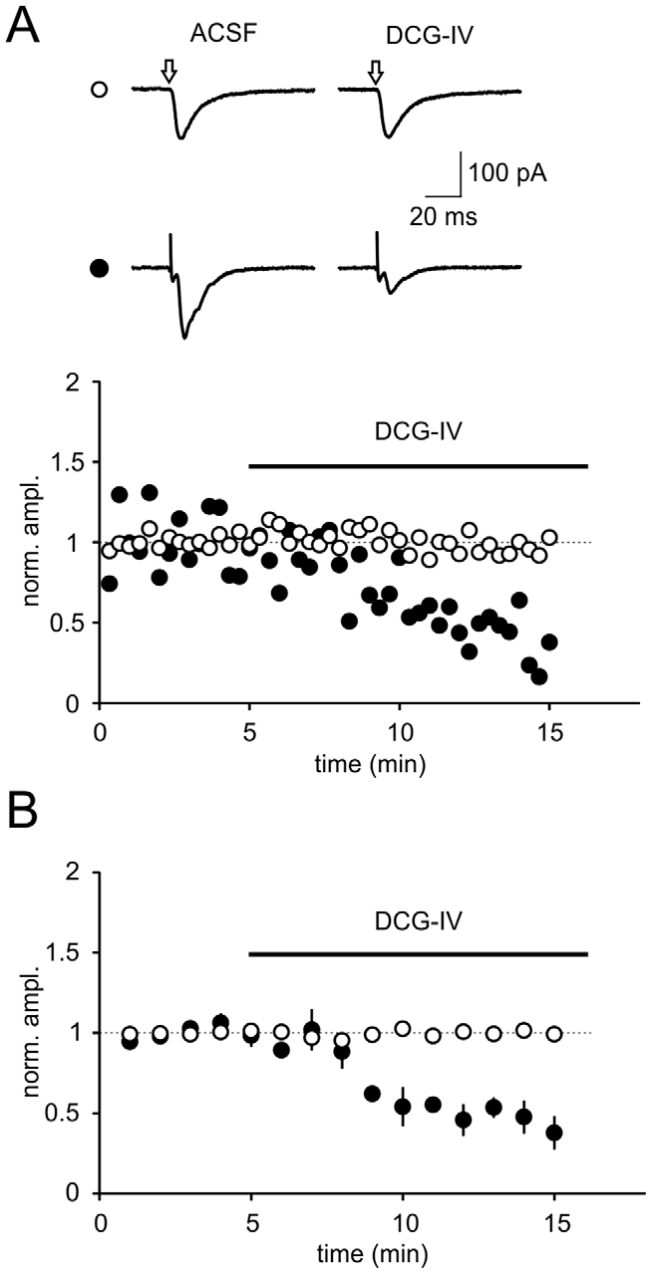
Glutamate uncaging responses were unchanged in DCG-IV. (A) A representative experiment for a burst firing neuron is shown. Glutamate was uncaged using a brief laser pulse (indicated with an open arrow; open circle), following synaptic stimulation (closed circle). DCG-IV reduced the synaptic response, the ‘uncaged’ response, however, was unchanged in the presence of DCG-IV. (B) Summary of all experiments (uncaging responses: n = 6; synaptic responses: n = 3). In those experiments, in which extracellular-evoked synaptic and uncaging responses were recorded simultaneously (n = 3), APV (50 µM) was added to the bath solution to block NMDA receptors. Please note that no obvious difference in synaptic depression by DCG-IV was found compared to control conditions (see Figure 3).

Next, we performed additional field potential recordings using DCG-IV, a different group II mGluR agonist, at a concentration of 1 µM. This concentration is known to compare well with 10 µM L-CCG-1 [23]. The NMDA receptor antagonist (APV, 50 µM) was added to the bath solution throughout the experiment to avoid NMDA receptors activation [24]. As expected fEPSPs in the subiculum were depressed by 1 µM DCG-IV to a similar extent as observed with 10 µM L-CCG-1 (Fig. 1C; 0.33±0.08, n = 5; p>0.05 compared to 10 µM L-CCG-1, Student’s t-test). The mGluR group II antagonist LY341495 (3 µM) fully reversed the suppression of fEPSPs in the SUB (Fig. 1C).

**Table 1:** Summary of the applied stimulation protocols to activate mGluRs.

Protocol	Pulse(s) analysed	ASCF	LY341495	p
**20 pulses at 1 Hz**	norm. 20th	1.60±0.10 (9)	1.76±0.16 (9)	>0.05
**180 pulses at 1Hz**	norm. 170th–180th	1.51±0.15 (4)	1.44±0.21 (4)	>0.05
**75 pulses at 5 Hz**	norm. 61th–75th	1.73±0.11 (3)	1.49±0.14 (3)	>0.05
**10 pulses at 100 Hz + test stimulus**	norm. test stimulus	0.95±0.14 (5)	0.83±0.21 (5)	>0.05
**200 pulses at 200 Hz +2 test stimuli**	norm. 1st test stimulus	1.37±0.10 (6)	1.36±0.09 (6)	>0.05
**200 pulses at 200 Hz +2 test stimuli**	PPD (2nd test stimulus/1st)	0.67±0.04 (6)	0.68±0.07 (6)	>0.05

PPD: paired-pulse depression.

* paired t-test, one-tailed.

**Figure 7:**
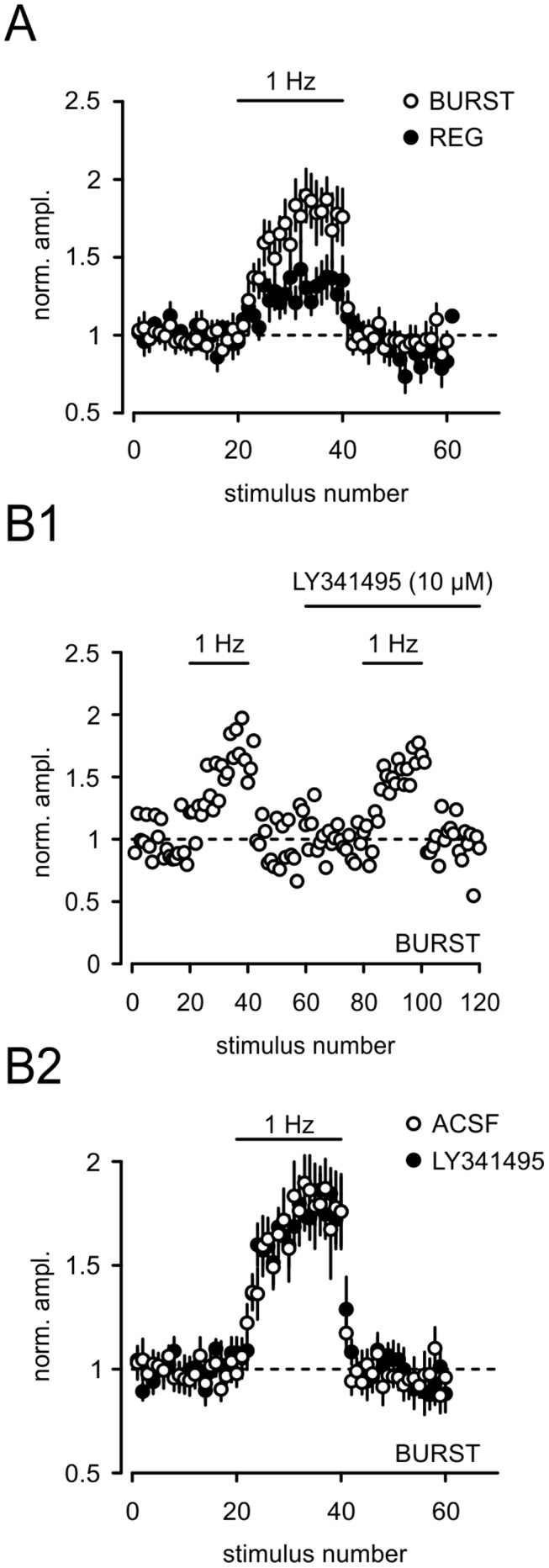
Frequency facilitation is not limited by activation of mGluRs. (A) Bursting and regular firing cells exhibited frequency facilitation. Changes in stimulation frequency from 0.05 Hz to 1 Hz (20 stimuli) resulted in a reversible facilitation of EPSCs. (B1) A typical experiment illustrating that the group II mGluR antagonist LY341495 (10 µM) did not have an effect on frequency facilitation in bursting cells. The results for nine such experiments (ACSF and LY341495) are summarized in (B2).

We then asked whether the inhibition of field potentials could also be ascribed on a cellular level to a particular cell type in the subiculum. We recorded from subicular pyramidal neurons and classified cells by their intrinsic firing pattern. Upon depolarization subicular pyramidal cells fire either a burst of action potentials or a train of single action potentials (Fig. 3A1). A burst of action potentials is typically defined as 2–4 action potentials occurring at a frequency of above 100 Hz [25]–[28].

In the presence of the GABA_A_-receptor antagonist gabazine (1 µM) we recorded excitatory postsynaptic currents (EPSC) at a holding potentials of –60 mV. After recording a stable baseline we bath-applied 1–2 µM DCG-IV. We observed a highly significant reduction of evoked EPSCs in burst firing neurons (to 45±6% of baseline levels, p<0.001; control: 374±21 pA vs. DCG-IV: 167±17 pA, p<0.001, n = 7, Fig. 3A2 and 3B). Interestingly, only a small reduction in the EPSC amplitude was found in regular firing cells (to 83±5% of baseline levels; control: 343±30 pA vs. DCG-IV: 279±19 pA, p>0.05, n = 5, Fig. 3A2 and 3B). It is important to note that the differences between bursting and regular firing neurons in response to DCG-IV are highly significant (BURST: n = 7; REG: n = 5; p<0.01, Student’s t-test).

### Presynaptic Site of Action

Next, to determine whether the reduction of the EPSC amplitude is due to a pre- or postsynaptic effect, we examined alterations in the paired-pulse ratio (PPR) in bursting neurons. For this purpose, afferent fibers were stimulated in close succession with an interstimulus interval of 50 ms. Changes in PPR are indicative of a presynaptic site of action [29]. Figure 4A shows a typical example of the evoked EPSCs and the accompanying changes in PPR. On average the PPR was increased by 20–30% (Fig. 4B, p = 0.03, n = 7).

The analysis of the coefficient of variation is an additional method to differentiate between pre- and postsynaptic alterations in synaptic strength [30]. As illustrated in Fig. 4C, this analysis also provided evidence for a presynaptic expression mechanism of DCG-IV induced suppression of EPSCs in burst firing neurons. The estimated values for CV^2^(control)/CV^2^(DCG-IV) plotted against the ratio of the EPSC(DCG-IV)/EPSC(control) fell close the bisecting line consistent with a presynaptic effect of DCG-IV [30].

To further prove the presynaptic site of action of the group II mGluRs, we performed two additional sets of experiments at the CA1– burst firing neuron synapse.

First, a minimal stimulation protocol was applied. In this experiment the failure ratio directly correlates with the release probability and is therefore a good measure for presynaptic changes. The stimulation strength was adjusted stepwise to a threshold where quantal-like EPSCs could be evoked and were alternating with failures, indicating the stimulation of a single axonal fiber. After recording a stable EPSC/failure ratio for at least 10 minutes 1 µM DCG-IV was applied. The agonist led to a significant increase of the percentage of failures as compared to baseline conditions (Fig. 5, ACSF: 34±5%, DCG-IV: 73±8%, n = 6; p = 0.002). Second, uncaging of glutamate was used to activate postsynaptic glutamate receptor on bursting neurons. Wash-in of DCG-IV did not lead to a reduction of postsynaptic currents evoked by glutamate uncaging (Fig. 6A and B: open circles, n = 6), whereas simultaneously recorded EPSCs evoked by synaptic stimulation were reduced by more than 50% (Fig. 6A and B: closed circles, n = 3, DCG-IV: 38±10% of baseline values, in the presence of 50 µM D-APV).

In summary, these experiments provide further evidence for a presynaptic mechanism of mGluR-mediated suppression of excitatory transmission onto subicular bursting neurons.

### Frequency Facilitation

Presynaptic mGluRs have been shown to be activated in a use-dependent manner. Under low-frequency stimulation synaptically released glutamate usually does not activate presynaptic receptors. However, prolonged presynaptic activity, which increases synaptic glutamate concentrations, is sufficient to activate presynaptic mGluRs [11]. As shown in Figure 7 CA1-SUB fibers were stimulated with a low frequency of 0.05 Hz and then at 1 Hz for 20 stimuli. We, first, observed a significant difference in frequency facilitation between both cell types (BURST: 1.80±0.16, 11^th^–20^th^ stimulus normalized to baseline, n = 10; REG 1.31±0.10, n = 7; p<0.05, Fig. 7A). Next, we wanted to know whether mGluRs act as autoreceptors onto bursting cells limiting synaptic transmission during repetitive stimulation. Figure 7B shows a representative experiment in which we applied the group II mGluR antagonist LY341495 (10 µM). Noteworthy, application of LY341495 has no effect on baseline synaptic transmission (Fig. 7B1) and frequency facilitation (20 pulses at 1 Hz; ACSF: 1.60±0.10, n = 9; LY341495∶1.76±0.16, p>0.05, 20^th^ stimulus normalized to baseline, n = 9, Fig. 7B1 and B2). Additionally, neither prolonged 1 Hz stimulation with 180 stimuli (3 minutes), nor 5 Hz stimulation with 75 stimuli resulted in a significant increase in the EPSP amplitude in the presence of LY341495 (1 Hz: ACSF: 1.51±0.15, n = 4; LY341495. 1.44±0.21, n = 4; p>0.05, 170^th^ –180^th^ stimuli normalized; 5 Hz: ACSF: 1.73±0.11, n = 3; LY341495. 1.49±0.14, n = 3; p>0.05, 61^st^ –75^th^ stimuli normalized; see Table 1). Furthermore, we investigated if high frequency burst were able to activate presynaptic mGluRs. We applied a burst of 10 pulses at 100 Hz followed by a test stimulus with a delay of 200 ms (5 times, 20 s apart). However, this stimulation protocol also failed to reveal differences between the two groups (ACSF: 0.95±0.14, n = 5; LY341495∶0.83±0.21, average of test stimulus normalized to baseline; n = 5; p>0.05).

It has been shown that the action of presynaptic group III mGluRs can be masked by a passive equilibration of the number of presynaptic release sites and the release probability during repetitive stimulation [31]. To test whether this is also true for presynaptic group II mGluRs we repeated the same experiment and applied a 200 Hz stimulus for 1 s with 2 test stimuli (ISI 50 ms) 2 s apart. Neither the 1st test stimulus (ACSF: 1.37±0.10, n = 6; LY341495∶1.36±0.09; n = 6, average of test stimulus normalized to baseline), nor the paired pulse ratio of both stimuli (ACSF: 0.67±0.04; LY341495 = 0.68±0.07; n = 6) showed any difference between both conditions. In summary, neither prolonged low-frequency stimulation, nor high frequency bursts are able to activate presynaptic mGluRs (see Table 1).

## Discussion

Here, we have shown that excitatory synaptic transmission at CA1-SUB synapses can be modulated by a group II mGluR agonist in a target-cell specific manner. Consistent with previous findings, Schaffer collateral synapses are not targeted by group II mGluRs [18], [19], and neither are fibers onto regular firing cells in the subiculum. Furthermore, analyzing the paired-pulse ratio, CV^−2^ and failure rates we provide evidence that the group II mGluR agonist DCG-IV suppresses synaptic transmission onto burst firing neurons via a presynaptic mechanism. Glutamate uncaging techniques confirmed the presynaptic origin. However, increasing synaptic glutamate concentration by frequency facilitation does not lead to activation of presynaptically located mGluRs.

In contrast to ionotropic glutamate receptors, which mediate fast neurotransmission in the brain, mGluRs often modulate synaptic activity by different second messenger cascades. When located postsynaptically mGluRs primarily modulate intrinsic conductances, whereas presynaptically located mGluRs control neurotransmitter release from the terminal [32], [33]. To the best of our knowledge, this is the first time that functional properties of group II mGluRs have been investigated in the SUB. We demonstrate that presynaptic mGluRs could be activated pharmacologically with two different drugs, DGC-IV and L-CCG-1. Group II mGluRs (mGluR 2 and 3) located in the terminals exert their action by inhibiting cAMP pathways. Previously, we have shown that an increase of cAMP is accompanied with an LTP protocol in bursting cells [34].

Activation of group II mGluRs may also regulate synaptic plasticity in the SUB, similarly to the hippocampal mossy fiber, where group II mGluRs agonists have been shown to induce long-lasting depression (LTD) of synaptic transmission [35]. However, in bursting neurons low-frequency stimulation at 1Hz with 900 pulses has been shown to induce NMDA receptor-dependent LTD [36]. Future studies will have to address the question whether a form of mGluR-dependent plasticity also exists in the SUB.

The subiculum occupies a central position within the hippocampal formation making it ideal for controlling and modulating hippocampal output. Interestingly, the subiculum contains two electrophysiologically distinct principal cells, regular and burst firing cells [37], [38]. The present study demonstrates that the activation of presynaptic group II mGluRs inhibits glutamatergic transmission selectively onto bursting neurons in the subiculum. These observations constitute further evidence that these two cell types within the subiculum represent two functional units which may have different roles in processing information [34], [36], [39], [40] as, intriguingly, both cell types have been shown to target different cortical and subcortical areas [41]–[43].

The group II mGlu-mediated depression of excitation may have a number of physiological roles. First, it is likely to be important for preventing massive excitation of bursting neurons in the SUB. It has been shown that the SUB is indeed involved in the generation of interictal discharges in vitro [3], [5] and in vivo [6]. Even though activation of bursting neurons has been shown to be tightly controlled by local inhibition [44], one might speculate that the mGluR-mediated negative feedback mechanism may be dysfunctional under chronic epileptic conditions. The consequence would be massive neuronal excitation, mainly in bursting neurons. As a cell loss has been described in the SUB under chronic epileptic conditions, in parallel with an inversed cell ratio of bursting to regular firing cells, bursting neurons may selectively die due to hypothesized hyperexcitability [45]. Therefore, it is of profound interest to investigate the actions of mGluR activation in rodent models of neurological and psychiatric disorders, which are paralleled by neuronal hyperexcitability and misbalanced neuronal transmission in the SUB [14], [15].

In summary, future studies have to further clarify the roles of group II mGluRs under physiological and pathological conditions. Additionally, it will be of interest whether other mGluRs also modulate synaptic transmission in the SUB, the main output region of the hippocampus.
